# Supplementary material for: Sorting at embryonic boundaries requires high heterotypic interfacial tension
Source: Nat Commun. 2017 Jul 31;8:157. doi: 10.1038/s41467-017-00146-x (PMC5537356; doi:10.1038/s41467-017-00146-x)
Supplement: Supplementary file 2 — Supplementary Software 1 [file 41467_2017_146_MOESM2_ESM.zip › PottsModel/SrcPottsModel/doc/model/class-use/Constants.HeatMapParameter.html]

Uses of Class model.Constants.HeatMapParameter


---


|  |  |  |
| --- | --- | --- |
| **Overview** \| **Package** \| **Class** \| **Use** \| **Tree** \| **Deprecated** \| **Index** \| **Help** | |  |
| PREV   NEXT | **FRAMES**    **NO FRAMES**     **All Classes** |  |


---


## **Uses of Class model.Constants.HeatMapParameter**

| Packages that use Constants.HeatMapParameter | |
| --- | --- |
| **model** |  |

Uses of Constants.HeatMapParameter in model

| Fields in model declared as Constants.HeatMapParameter | |
| --- | --- |
| `protected  Constants.HeatMapParameter` | `Pixel.cHeatMapParam` |
| `Constants.HeatMapParameter` | `Constants.ParameterManager.heatMapParam` |

| Methods in model that return Constants.HeatMapParameter | |
| --- | --- |
| `static Constants.HeatMapParameter` | `Constants.HeatMapParameter.valueOf(java.lang.String name)`             Returns the enum constant of this type with the specified name. |
| `static Constants.HeatMapParameter[]` | `Constants.HeatMapParameter.values()`             Returns an array containing the constants of this enum type, in the order they are declared. |

---


|  |  |  |
| --- | --- | --- |
| **Overview** \| **Package** \| **Class** \| **Use** \| **Tree** \| **Deprecated** \| **Index** \| **Help** | |  |
| PREV   NEXT | **FRAMES**    **NO FRAMES**     **All Classes** |  |


---
